# Tool-stone selection in the African Middle stone age at Sibhudu cave

**DOI:** 10.1371/journal.pone.0350817

**Published:** 2026-06-04

**Authors:** Patrick Schmidt, Klaus G. Nickel

**Affiliations:** 1 Petrology and Mineral Resources, Department of Geosciences, Eberhard Karls University of Tübingen, Germany; 2 Archaeometry, Department of Prehistory, Early History and Medieval Archaeology, Eberhard Karls University of Tübingen, Germany; 3 Department of Archaeology, University of Cape Town, Rondebosch, South Africa; 4 Applied Mineralogy, Department of Geosciences, Eberhard Karls University of Tübingen, Germany; 5 Eberhard Karls University of Tübingen, Competence Center Archaeometry – Baden Wuerttemberg, Tübingen, Germany; Universita degli Studi di Ferrara, ITALY

## Abstract

The raw materials people used for making stone tools may contain information about their territory, exchange routs or the selection criteria they employed during provisioning. In this study, we measure the mechanical properties of different tool-stones used by foragers living during the Middle Stone Age at Sibhudu Cave in South Africa. The site yielded a long and continuous sequence that saw transitions between different raw materials and tool forms. We evaluate the quality of these different stones for tool making and use, attempting to find correlations between selected raw materials and the tools made from them. We find that the raw materials used at Sibhudu have substantially different qualities, some being easy to flake but weak upon use, some being tough during stone knapping and resistant during use. Comparing these data with the appearance and disappearance of tool types throughout the Sibhudu sequence, we note that tool-stones requiring lower flaking forces were more often retouched than those requiring great forces. Elongated products, blades, were mostly made from materials with better fracture predictability, suggesting an understanding of the basic requirements for standardising the tool knapping process. Use-related qualities, such as resistance to dulling, appear to have been of lesser importance at Sibhudu. Our results suggest that the site’s occupants had a good understanding of the qualities of rocks for specific knapping processes.

## Introduction

The African Middle Stone Age (MSA) has seen major innovations, regional patterns in the stone toolkit [1] and new human forms. In southern Africa, the second half of the MSA is associated with *Homo sapiens* [2]. During this period, discrete techno-cultural entities build a diverse sequence with sub-stages identifiable across the subcontinent [3]. These sub-stages were also associated with major transitions in the raw materials used for tool making [4]. It has been argued that raw material procurement patterns reflect the territories of foragers [5,6]. Others proposed that raw material choices were driven by the need for certain tool-stones and the knowledge of the mechanical properties that are useful for specific tool types [7]. Measuring the mechanical properties of raw materials may help understand which of these two scenarios was more likely to be true [8,9]. However, the comparison of different materials can only be conducted at archaeological contexts, which yielded long and continuous sequences and where different raw material types occur throughout the sequences. In South Africa, such archaeological sequences are rare. One example is Diepkloof Rock Shelter on the western seaboard [10]. There, it has recently been argued that foragers selected specific tools-stones to manufacture stone tools [8]. So far, Diepkloof provides the only available data set on mechanical properties of raw materials in the MSA. Another site with a long and continuous sequence is Sibhudu Cave (henceforth only Sibhudu) on South Africa’s eastern seaboard [11]. The site yielded a sequence from the pre-Stillbay (SB) to the post-Howiesons Poort (HP), where transitions of different raw materials can be tracked through time [12]. In this study, we analyse the mechanical properties of tool-stones found in the Sibhudu sequences. For this, we employ an approach based on standard measurements commonly employed in engineering [for an overview see for ex.: 13]. The measurement of standard mechanical properties, such as strength, fracture toughness or hardness, has been widely applied to archaeological stone raw materials from different parts of the world [e.g., 14, 15–17]. Standard quantities provide comparable data across different contexts and researchers. However, a clear link to what archaeologists call knapping quality was missing until recently. Nickel and Schmidt [9] proposed a theoretical framework allowing to combine the standard measurements fracture toughness, strength, hardness and stiffness to make predictions on the force needed to induce flaking. This allows comparing knapping force requirement in different tool-stones, establishing an objective ranking of different materials. The absolute values of such force predictions must be regarded as approximations of the force requirements in real world knapping because they are based on a fracture initiation mechanism (mode I fracture opening by indentation) that is different from that in stone knapping (i.e., Hertzian fracture). However, values obtained on different samples are comparable and the absolute values are overall not too far off from the force requirements in real stone knapping (compare for example the knapping force values in [18] and [8]). Here we apply the refined model proposed by Schmidt et al. [8] to make such force predictions. Another measurable aspect of knapping quality has been proposed to be the predictability of the raw materials’ fracture behaviour [19,20]. We measure fracture predictability with an approach based on Weibull’s [21] fracture theory. The advantage of such Weibull statistics over the more traditionally used ‘impurity encounter rate’ [19] is that the approach provides comparable values derived from actual tests of the failure behaviour of rocks. Both these measures, fracture force and predictability, also allow making statement on the durability of tools when used as projectiles or for other impact-based use types (in other words the resistance against impact fractures). To widen our predictions made on the durability of different tool-stones, including repetitive use types (scraping, cutting), we also conduct abrasion tests of the Sibhudu raw materials. Combining these three quantitative approaches, we hope to explain some of the raw material selection criteria active during the last part of the MSA on South Africa’s eastern seaboard.

## Methods and materials

### Samples

We analysed six samples representing the major tool-stone classes used at Sibhudu. Those are hydrothermal quartz (OK-21–01), sandstone/quartzite (OK-21–03 and −05), dolerite (OK-21-04a and -04b) and hornfels (OK-21–18). The rock wall of Sibhudu (WK-21–03) is made of arkosic sandstone of the Natal Group [22]. Quartzites were those found in the uThongathi River in front of Sibhudu (sample −05). Dolerite was also sampled in the uThongathi River, hydrothermal quartz from a dry river bed approximately 100 km to the south and hornfels from a primary outcrop approximately 150 km to the north of the site. All samples were made into standard bending bars with chamfered edges. Bending bar dimensions are reported in S1 Table. Sample descriptions are summarised in Table 1.

**Table 1:** List of samples, descriptions and predicted knapping force (*F*_*ac*_).

Sample N°	Rock	Description	*F*_*ac*_ in 30 mm^3^ [N]	*W*_*s*_ [mm^3^/Nm]
**OK-21–01**	Hydrothermal quartz	Rolled white cobble. Sampled on a terrace of the Mpambanyoni river (30°16’24.1“S 30°42’19.1”E), ~ 100 km SW of Sibhudu.	428	5.09 x 10^−3^
**OK-21–03**	Sandstone	From the rock wall of Sibhudu Cave, sometimes called “Sibhudu sandstone”.	754	3.42 x 10^−3^
**OK-21–05**	Quartzite	Rolled cobble from the uThongathi river, sampled in front of Sibhudu Cave.	4507	5.08 x 10^−3^
**OK-21-04a**	Dolerite	Rolled cobble from the uThongathi river, same location as OK-21–05.	3808	7.52 x 10^−3^
**OK-21-04b**	Dolerite	Rolled cobble from the uThongathi river, same location as OK-21–05.	1719	7.14 x 10^−3^
**OK-21–18**	Hornfels	Samples in primary position near Ndindindi (28°8’28.63“S 30°38’53.15”E), ~ 150 km NW of Sibhudu.	43	9.63 x 10^−3^

Values of *F*_*ac*_ are given for samples with a volume of 30 mm^3^ (for an explanation of this volume, see main text).

### Instruments and data treatment

We performed four-point-bending experiments with an Instron 4502 10kN universal testing machine. During these tests, bending bars were loaded (until failure) on their tensional side with a speed of 1 mm/min (after a preloading time of 10 N). Upper and lower bearing distances were 20 and 40 mm. All measurements reported are from bending bars that broke within the limits of the two upper bearings (roughly in the middle of the bars). Values obtained from bars that broke outside of the bearings were discarded. Fracture strength (*σ*_*f*_) for each bar was calculated as


$$\begin{array}{c} \sigma_{f}=\;\frac{\text{3}}{\text{2}}\frac{F(S_{\text{1}}{-S}_{\text{2}})}{{bh}^{\text{2}}} \end{array}$$ (1)

where *F* is the force at failure, as recorded by the testing machine, *b* and *h* the width and height of the bending bars and *S*_1_ and *S*_*2*_ the span of the lower and upper bearings, respectively (here 40 and 20 mm). These strength values were then plotted using Weibull’s [21] method. Weibull plots are double logarithmic plots of the failure probability ($\text{ln}\left(ln\left(\frac{\text{1}}{\text{1}-F_{i}}\right)\right)\text{with }F_{i}=\frac{\sigma_{f}-\text{0}.\text{5}}{number\;of\;tests}$) over strength (ln (*σ*_*f*_)) [21]. They result in a characteristic strength value (*σ*_*0*_) and the Weibull modulus (*m*), a measure of the homogeneity of the fracture behaviour across all tested bars. In these plots, *σ*_*0*_ is calculated as the point of intersection of the linear best fit of the data and the abscissa and *m* as its slope. We take *m* as an approximation of the samples’ fracture predictability in a real-world knapping situation. The effective volume *V*_*0*_ to which *σ*_*0*_ applies is calculated as


$$\begin{array}{c} V_{\text{0}}=\frac{m+\text{2}}{\text{4}(m+\text{1}{)}^{\text{2}}}\cdot S_{\text{1}}\cdot b\cdot h \end{array}$$ (2)

Indentation fracture resistance (*K*_*Ic*_), an approximation of fracture toughness, was measured using the same testing machine and Vickers diamonds (load = 100 N, hold time = 30 s). Each sample was indented multiple times in a diamond-polished surface to obtain a statistically representative number of indentation cracks. *K*_*Ic*_ was calculated from the mean length of these cracks (*c*) using Niihara et al.’s [23] *K*_*Ic*_ relation reformulated to enter the full diagonal of the indentation negative (*a*_*1*_) instead of the half diagonal:


$$\begin{array}{c} K_{Ic}=\text{0}.\text{0}\text{1}\text{6}\text{7}\text{5}\;\frac{\;{a_{\text{1}}}^{\text{2}}\;{HV}^{\text{3}/\text{5}}\;E^{\text{2}/\text{5}}}{c^{\text{3}/\text{2}}} \end{array}$$ (3)

where *HV* is Vickers hardness and *E* Young’s modulus [the rationale for using the full diagonal can be found in 8]. Young’s modulus was measured in flexural vibration mode using an IMCE (Belgium) RFDA unit and a frequency range between 0 and 100 kHz. As the calculation of *E* requires the material’s Poisson’s ratio, we admitted it to be equal to 0.17 (as for silica glass) [24].

As proposed by Schmidt et al. [8], we calculated detachment force (in N) as


$$\begin{array}{c} F_{ac}=\text{5}.\text{8}\text{0}\text{4}*{\text{1}\text{0}}^{\text{6}}\;\frac{\;{K_{Ic}}^{\text{4}}{\left(\frac{HV}{E}\right)}^{\text{2}/\text{5}}}{{\sigma_{\text{1}}}^{\text{3}}} \end{array}$$ (4)

where *σ*_*1*_ is the strength of a given volume that can be calculated from *σ*_*0*_, as obtained from Weibull plots, using


$$\begin{array}{c} \sigma_{\text{1}}=\sigma_{\text{0}}\sqrt[{m}]{\frac{V_{\text{0}}}{V_{\text{1}}}} \end{array}$$ (5)

where *V*_*1*_ is the volume for which *F*_*ac*_ is to be calculated.

Because *F*_*ac*_ depends on volume, its magnitude will change for the same tool-stone in different knapping situations (e.g., near edge flaking vs. knapping blows further away from the edge). A broader value describing tool-stones is therefore the integral of the *F*_*ac*_/volume function over a given range of volumes. If we admit a realistic range of the volumes active during knapping as 1–600 mm^3^, this integral is (in Nmm^3^)


$$\begin{array}{c} C\int_{\text{1}}^{\text{6}\text{0}\text{0}}{V_{\text{1}}}^{\text{3}/m}\;dV_{\text{1}} \end{array}$$

with


$$\begin{array}{c} C=\text{5}.\text{8}\text{0}\text{4}*{\text{1}\text{0}}^{\text{6}}\;\frac{\;{K_{Ic}\left(\frac{HV}{E}\right)}^{\text{2}/\text{5}}}{{\sigma_{\text{0}}}^{\text{3}}\;{V_{\text{0}}}^{\text{3}/m}} \end{array}$$

To convert this to SI units, here Nm^3^, the result is multiplied by 10^−9^.

We measured abrasion resistance with a pin-on-disc tribometer, and 80-grit SiC sandpaper as rotating phase (for each measurement, a new sheet was used). Load was 10N. These experimental conditions were not chosen to represent real-world use of stone tools but rather to provide repeatable standard conditions comparable to previously published data [8]. The linear speed under the sample pins was 1 m/s, and ~300 m of sandpaper passed under each sample in 5 min measuring time. We measured three pins of each sample and then averaged the volumes lost by each pin during the tests (as calculated from the mass and the samples’ density in S1 Table). Specific wear rate (*W*_*s*_) was calculated as


$$\begin{array}{c} W_{s}=\frac{\Delta V}{FD} \end{array}$$ (6)

where Δ*V* is the lost volume of the pins and *D* the distance of the sandpaper that passed under the pins during the test.

## Results

**Fig 1:**
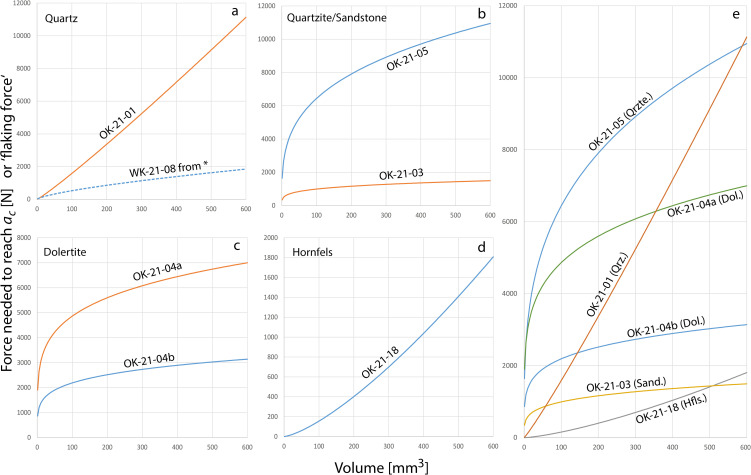
Volume dependence of *F*_*ac*_, the force predicted to reach critical crack length *a*_*c*_. Note that ordinate scales are different in the four plots (a to d) for better visibility of the curvature of the functions. *The dotted line in (a) is generated from hydrothermal quartz data in [8] for comparison. Plot (e) shows all data of this study together for better comparability.

All measured mechanical properties are summarised in Table 1 (*F*_*ac*_ and *W*_*s*_) and Table 2 (all other values) and Fig 1. Table 3 reports integrals of the *F*_*ac*_/volume functions. Raw data are given in S2-S4 Tables and S1 Fig. Four-point-bending data obtained on hydrothermal quartz sample OK-21–01 are fragmentary. Only three bending bars yielded values of *E* and only seven of the bars broke between the upper two bearings. Therefore, we also refer to the values of hydrothermal quartz of Schmidt et al. [8] to ascertain that our values for hydrothermal quartz are not totally off. Hydrothermal quartz and hornfels have the lowest Weibull moduli, indicating that they have the lowest fracture predictability of all analysed rocks (our quartz *m* value of 2.8 is roughly consistent with *m* = 4.3 in Schmidt et al. 2024). Dolerite has the highest Weibull modulus of all samples, suggesting a good fracture predictability in real-world knapping situations. The Sibhudu rock wall sandstone comes close to this fracture predictability value. Hornfels requires by far the lowest force for flake detachment (~100x less force than our quartzite sample from the river at 30 mm^3^ and ~10x less over the volumetric range from 1 to 600 mm^3^). Quartzite and dolerite from the uThongathi River require the highest flaking forces. The second lowest flaking force at 30 mm^3^ was measured in hydrothermal quartz (our value for 30 mm^3^ is approx. double that of hydrothermal quartz in Schmidt et. 2024). However, the relatively great magnitude of the 1–600 mm^3^ integral documents that low flaking forces are only required if hydrothermal quartz is worked in small volumes. At greater volumes, this tool-stone requires similar forces to quartzite and Dolerite. Sandstone from the Sibhudu wall presents an apparently good trade off, requiring relatively low fracturing force but having relatively good fracture predictability (it requires between 5 and 6 times less force than quartzite and dolerite from the uThongathi but it has the second best fracture predictability).

**Table 2:** Mechanical properties as experimentally determined on the six rock samples.

Sample N°	Density [g/cm^3^]	*E* [Gpa]	Mean *c* [µm]	*K*_*Ic*_ [MPa* √m]	*σ*_*f*_ [MPa]	*m*	*V*_*0*_ [mm^3^]	*σ*_*0*_ [MPa]
**WK-21-08b (Qrz.)**	2.50	61.18	280	1.46	14.34	2.8	189	16.20
**OK-21–03 (Sand.)**	2.54	49.53	258	1.33	20.12	13.1	158	21.04
**OK-21–05(Qrzte.)**	2.53	67.40	176	2.67	28.68	10.1	55	30.11
**OK-21-04a (Dol.)**	2.92	95.15	192	4.51	52.03	14.8	38	53.87
**OK-21-04b (Dol.)**	2.92	94.11	191	4.51	61.01	14.9	185	63.30
**OK-21–18 (Hfls.)**	2.59	80.58	235	2.84	53.89	2.2	237	60.89

*K*_*Ic*_ was calculated from the mean crack length *c*. Here, *σ*_*f*_ is the average strength of all bending bars of one sample. Weibull modulus *m* and characteristic strength *σ*_*0*_ were determined from Weibull plots and *V*_*0*_ is the volume for which *σ*_*0*_ is valid. Qrz. = hydrothermal quartz, Qrzte. = quartzite, Hfls. = hornfels, Dol. = dolerite, Sand. = sandstone.

**Table 3:** Integrals of the *F*_*ac*_/volume functions.

Sample	Integral over 1–600 mm^3^ (Nm^3^)
OK-21–01 (hydroth. quartz)	3.20 x 10^−3^
Ok-21–03 (sandstone)	0.73 x 10^−3^
OK-21–05 (quartzite)	5.07 x 10^−3^
Ok-21-04a (dolerite)	3.49 x 10^−3^
Ok-21-04b (dolerite)	1.57 x 10^−3^
OK-21–18 (hornfels)	0.46 x 10^−3^

Abrasion resistance values of quartz bearing rocks hydrothermal quartz and quartzite lie close to 5 x 10^−3^ mm^3^/Nm. Sandstone has the best resistance to abrasion with 3.4 x 10^−3^ mm^3^/Nm. Values of hornfels and dolerite are substantially greater and lie above 7 x 10^−3^ mm^3^/Nm, with hornfels even being greater than 9 x 10^−3^ mm^3^/Nm. Based on these data, hornfels is the weakest to abrasion (weaker than sandstone by a factor of 3). However, it appears that dolerite presents the worst combination of the two properties knapping force requirement and durability. Only its good fracture predictability seems to make it an interesting raw material. The interesting combination of properties of Sibhudu wall sandstone is further highlighted by its relatively good resistance to abrasion (suggesting good durability of the tools in cutting situations).

## Discussion

Our data have been obtained on single blocks from each raw material class (except for dolerite, where we analysed two samples). This obviously bears the risk of oversimplifying the properties of these material classes. There is heterogeneity in what is usually called dolerite, hornfels or quartzite. Our values of the measured properties should therefore be considered as indications, rather than values representing all hornfels, or all dolerites. However, the comparison of our data with those obtained by previously published analyses [8,25] shows that values obtained on the same rock types are similar. Only in the future, when more and similar studies on the same rock types will be available, more precise statements on their heterogeneity will be possible.

**Fig 2:**
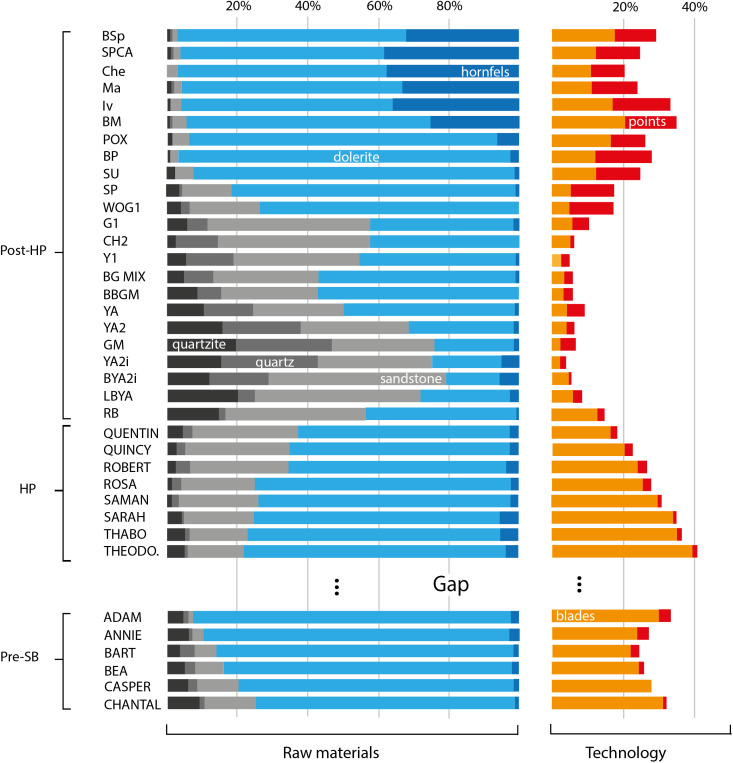
Raw material distributions in Sibhudu (only Conard excavations). Percentages of blades/bladelets and points were calculated to all pieces in the lithic assemblage. Data recalculated after [28–30], total lithic assemblage = 22992 (only artefacts > 30 mm).

However, our findings do allow explaining some of the raw material transitions found throughout the Sibhudu sequence. Fig 2 shows these transitions in the pre-SB, HP and post-HP layers excavated during the Conard excavations. Raw material data for the SB are still unpublished for the Conard excavations. Our argumentation will mostly rely on the sequence excavated during the Conard excavations, but we will sporadically make refence to SB data from the earlier Wadley excavations [26,27] to provide a more complete understanding of the Sibhudu sequence.

Throughout this sequence, there appears to be an association between the production of blade blanks and the use of dolerite (compare in Fig 2). The use of quartz-bearing raw materials seems to be correlated with the production of flake blanks. The association between dolerite and blade production can be well appreciated in the HP and the lower layers of the post-HP. There, the frequency of blades over flakes decreases with time. Concomitantly, backed pieces, which are normally made from blade blanks [31], occur less often [28]. Dolerite also decreases from bottom to top of this HP-to-lower-post-HP sequence. At the same time, bifacial points begin to appear in the upper part of the HP and the lower layers of the post-HP [30]. These points are mainly made on quartz-bearing rocks: in the early post-HP there are seven bifacial points, five made from hydrothermal quartz, one from chert (a raw material class that is anecdotal at Sibhudu) and one from quartzite [30]. In the upper HP layer, there are 12 bifacial points most of which are also made from hydrothermal quartz [28]. How can this transition be explained? Dolerite requires relatively high fracturing forces, even in small volumes, as highlighted by the hyperbolic shape of the *F*_*ac*_/volume curves in Fig 1c. High fracturing forces in small volumes render retouch and shaping difficult because stronger blows imply lower precision. Bifacial points obviously require shaping and retouch, so that hydrothermal quartz with its low *F*_*ac*_ at low volumes can be expected to be a superior raw material for making points, as compared to dolerite. On the other hand, hydrothermal quartz allows relatively bad fracture predictability. This is expected to be a problem for the production of elongated regular blanks where at least a minimum degree of standardisation is required if blanks are to be further transformed into specific end products (e.g., backed pieces). Thus, the transition across the HP and lowermost post-HP, away from dolerite to quartz bearing rocks in general, and the reduction of dolerite together with the increase of hydrothermal quartz used for bifacial points, appears to be the response to changing requirements in tool manufacture. Whether the association of retouch and tool-stones that require low fracturing forces is a real pattern can be verified on different parts of the Sibhudu sequence. The upper part of the sequence shows the strongest increase in hornfels. There is also a correlation between the frequency of retouched artefacts and hornfels in the upper part of the post-HP [32], i.e., hornfels was more retouched than other materials. This is perhaps best illustrated by a class of heavily retouched and re-sharpened cutting and/or scraping tools called Tongati tools [33] that appear in these layers. More than 60% of all points in these layers and most Tongatis, are made of hornfels [34]. Similarly, Wadley [26] found that hornfels was relatively more retouched than other materials in the SB at Sibhudu, where it was mostly used as scrapers. Thus, hornfels was used for pieces that required retouch in most cultural horizons at Sibhudu. We hypothesise that the reason for this was the low *F*_*ac*_ of hornfels, allowing more elaborate retouch than other rocks. However, explaining the higher rates of retouch on hornfels should take into account that it is most likely the raw material transported to Sibhudu over the longest distance [35]. Longer transport distances may influence the intensity of curation, and therefore retouch on the pieces [36]. The higher retouch frequency on hornfels might therefore reflect transport cost and curation along other factors, such as mechanical properties. A clearer picture emerges when considering dolerite use in the SB. Wadley [26] notes that dolerite was relatively less used to make bifacial points than for unretouched flake and blade blanks. This further strengthens the hypothesis that rocks, which require greater knapping forces were less chosen for retouched pieces. At first glance, this hypothesis does not appear to be supported by the finding of Soriano et al. [27] that SB dolerite bifacials were made at Sibhudu, as illustrated by many shaping flakes, but that the points themselves are missing. Thus, Sibhudu foragers did select dolerite for making bifacials, even though it is difficult to reliably estimated their exact percentage. However, this absence of finished points at the site may indicate that dolerite points broke more often and were not brought back to the site as often as points made from other materials. This would be confirmation of dolerite being less well suited to make durable bifacial points. Only more data on what happened to the Sibhudu dolerite points can shed light on these questions. As it standns, Wadley [26] reports ~50% of points being made from Dolerite. This shows that associations of over- and underrepresentation of specific raw material types and their mechanical properties are far from being linear. Relative abundances of raw materials were most likely driving by a combination of factors, quality for knapping and use being only two of those factors.

Concerning the usability of different raw materials, the Sibhudu sequence does not allow making clear statements, other than that it might have been a criterion of lesser importance. The case of bifacial points and scrapers in the SB suggests that the resistance during repeated use types might not have been too important a factor in tool-stone selection. Residue analysis on some of the points found in SB layers shows that they were used as butchery knives [37]. At the same time, residue and use-trace on Still Bay points show that some could have been used as spearheads [26]. Thus, retouched bifacial points might have been intended to be used for both cutting and projectile use. The two most represented raw material classes used for making these points in the SB were dolerite and quartzite. Both show very good resistance against the formation of impact fractures. Dolerite, however, is relatively weak against abrasion. Dolerite is relatively underrepresented in points, as compared to the overall SB assemblage [26], although a somewhat higher proportion of shaping flakes [27] might indicate that the actual number of dolerite point might have been slightly higher. At first sight, this might be understood as indicating that the main purpose of these points was projectile use. However, the overrepresentation of hornfels in scrapers (hornfels has a similar weakness against abrasion as dolerite) rather suggests that the intended use type was not the major driving force of tool-stones at Sibhudu. This is supported by the dominant choice of hornfels for cutting tools in the upper part of the sequence.

## Conclusion

It is not the intention of this study to explain every raw material transition as being the result of specific selection for mechanical properties. Humans are known to make complex choices that are sometimes guided by irrational reasoning and/or thought patterns. What our data show is that some of the materials were relatively more often used to make specific tools than others and that this might, in part, be explained by their mechanical properties. Examples for this are that hornfels and hydrothermal quartz were preferentially chosen for retouched tools, perhaps because they allow easy flaking, or that dolerite was chosen for elongated blanks (blades) because its good fracture predictability makes it more likely that the relatively long cracks needed to make blades run smoothly and undisturbed through the material. Other associations appear not explicable by the mechanical properties of tool-stones. The choice of hornfels for scraping and cutting tools, for example, appears counterintuitive because these rocks have relatively low resistance to abrasion. Only actualistic use tests may shed light on whether the specific tool-stones and forms show wear rates that are still acceptable in the particular use situations. As it stands, our study highlights the potential of quantitative measurements of the mechanical properties of tool-stones for making predictions on raw material choices of Stone Age foragers. It also shows that foragers living at Sibhudu had most likely a good understanding of the qualities of tool-stones for specific knapping processes.

## Supporting information

S1 Fig Weibull plots of the six samples analysed in this study. The formulae displayed are the linear best fits of the distributions of each plot, from which Weibull modulus *m* and characteristic strength *σ*_*0*_ values are extracted.
(TIF)

S1 Table Bending bar dimensions, stiffness and strength of all samples. Bending bars of each sample are ordered as a function of increasing *σ*_*f*_.
(XLSX)

S2 Table Crack lengths *c*, and the resulting indentation fracture resistance values *K*_*Ic*_. Crack lengths were measured from scanning electron microscope micrographs of Vickers indentations, of all samples.
(XLSX)

S3 Table Weibull modulus *m* and characteristic strength σ_0_. Values are obtained from the Weibull plots shown in S1 Fig. Values in brackets after the sample numbers are the numbers of bending bars analysed per sample to obtain Weibull modulus and characteristic strength values.
(XLSX)

S4 Table Masses as measured before and after pin-on-disc abrasion tests. Lost mass in cm^3^ was calculated using the densities reported in Table S1.
(XLSX)
