# Supplementary figures and images for: Tool-stone selection in the African Middle stone age at Sibhudu cave
Source: PLoS One. 2026 Jun 4;21(6):e0350817. doi: 10.1371/journal.pone.0350817 (PMC13235935; doi:10.1371/journal.pone.0350817)

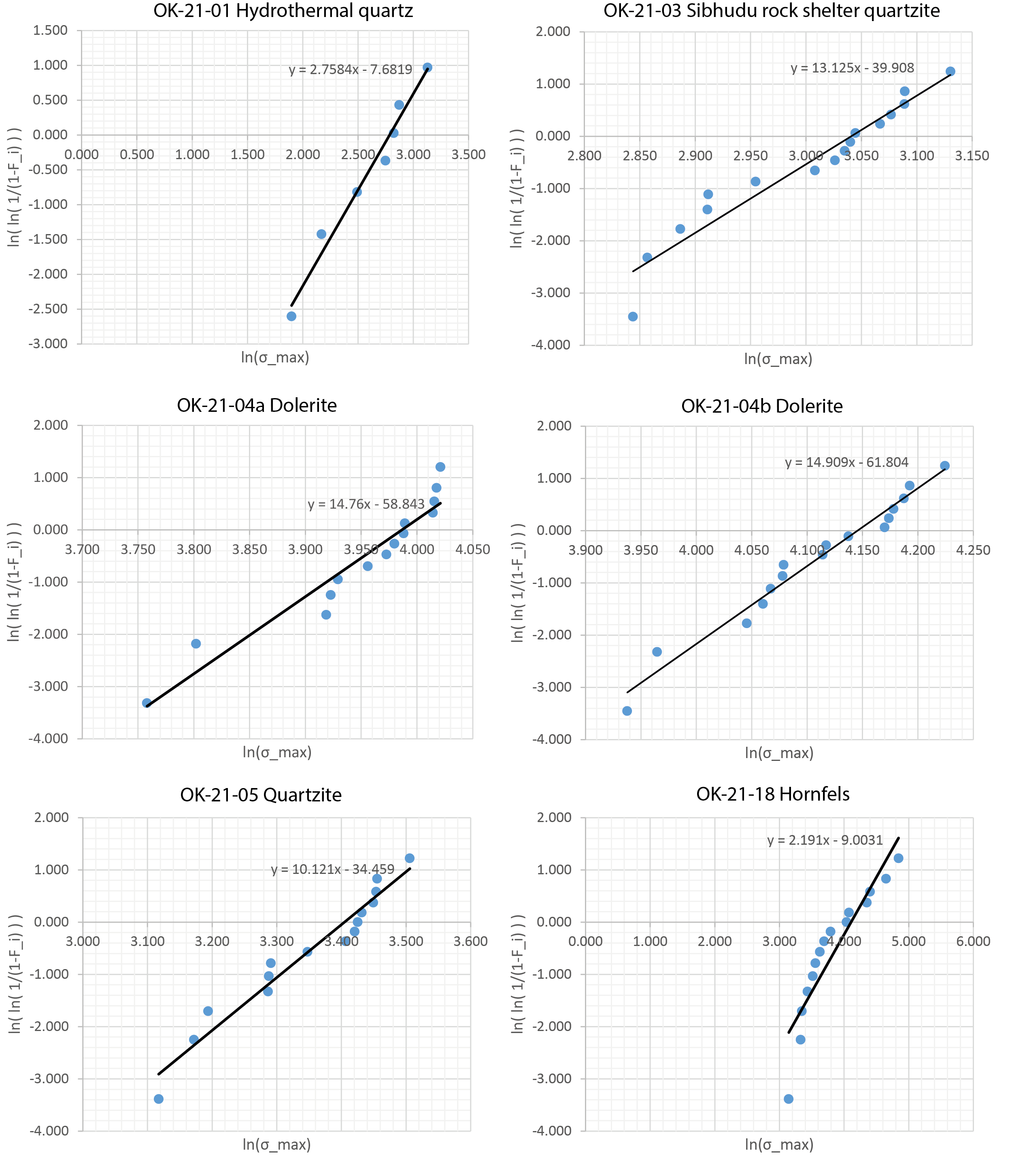

Supplement: S1 Fig — The formulae displayed are the linear best fits of the distributions of each plot, from which Weibull modulus m and characteristic strength σ0 values are extracted. (TIF) [file pone.0350817.s001.tif]
